# German translation, cultural adaptation and testing of the Person‐centred Practice Inventory – Staff (PCPI‐S)

**DOI:** 10.1002/nop2.511

**Published:** 2020-05-26

**Authors:** Maya L. D. Weis, Martin Wallner, Sabine Köck‐Hódi, Christiane Hildebrandt, Brendan McCormack, Hanna Mayer

**Affiliations:** ^1^ Department of Nursing Science University of Vienna Vienna Austria; ^2^ The Division of Nursing Queen Margaret University Edinburgh UK; ^3^ Centre for Person‐centred Practice Research Queen Margaret University Edinburgh Edinburgh UK; ^4^ University College of South‐East Norway Drammen Norway; ^5^ University of Pretoria Pretoria South Africa; ^6^ Maribor University Maribor Slovenia; ^7^ Ulster University Coleraine UK

**Keywords:** factor analysis, long‐term care, measurement, person‐centredness, psychometric analysis

## Abstract

**Aim:**

The aim of this study was to translate and culturally adapt the PCPI‐S into German and to eventually test its psychometric properties in long‐term care settings.

**Background:**

Person‐centred practice has been widely adopted internationally as a best‐practice model in nursing and health care. To ensure a sustainable implementation of this practice and to successively promote it, person‐centred practice should be evaluated on a regular basis. The Person‐centred Practice Inventory—Staff (PCPI‐S), which is based on McCormack & McCance's Person‐centred Practice Framework, is a new instrument for this purpose by assessing perceptions of person‐centredness among healthcare staff.

**Design:**

A two‐phase research design was used involving the translation and cultural adaption of the PCPI‐S from English to German (PCPI‐S‐G; Phase 1) and a quantitative cross‐sectional survey (Phase 2).

**Methods:**

Construct validity was evaluated using confirmatory factor analysis (CFA), and internal consistency was calculated using Cronbach's *α*.

**Results:**

Phase 1 was conducted using an internationally recommended checklist for translations and cultural adaptations. In Phase 2, the PCPI‐S‐G was tested in 15 residential care homes in Austria with a sample of 255 staff members. The CFA showed good construct validity and supported the theoretical framework. The internal consistency for the three constructs of the PCPI‐S was excellent, revealing Cronbach's *α*‐scores from 0.902–0.941.

## 1 INTRODUCTION

The concept of “person‐centredness” puts the person in the centre of the care process and decision‐making. The person is seen as a holistic, unique individual with their own choices, preferences and wishes, which must be considered to provide person‐centred care. The World Health Organization (2015, p.10–11) defines people‐centred health services as “…an approach to care that consciously adopts individuals’, carers’, families’ and communities’ perspectives as participants in and beneficiaries of, trusted health systems that respond to their needs and preferences in humane and holistic ways. […] [It also] requires that people have the education and support they need to make decisions and participate in their own care. It is organized around the health needs and expectations of people rather than diseases.” The formation and fostering of healthful relationships between all care providers, service users and others significant to them in their lives are of great importance in this concept (McCormack & McCance, 2017).

## 2 BACKGROUND

Person‐centredness is recognized as a best‐practice model in health care and has shown high relevance for health strategy (Jones et al., 2017; McCormack & McCance, 2017; Wilberforce et al., 2016). The concept has been implemented in practice, especially in the UK, Scandinavia, Canada, the USA and Australia and continues to internationally obtain interest as it underpins many western healthcare strategic developments (Bing‐Jonsson, Slater, McCormack, & Fagerström, 2018; McCormack & McCance, 2017). The implementation of a person‐centred practice philosophy has shown positive effects, not only for patients, but also for staff members and relatives (De Silva, 2014; Edvardsson, Sandman, & Rasmussen, 2008; Han, 2016; Hudon, Fortin, Haggerty, Lambert, & Poitras, 2011; Martinez, Suarez‐Alvarez, & Yanguas, 2016; McCormack & McCance, 2017; Sjogren, Lindkvist, Sandman, Zingmark, & Edvardsson, 2012; Wilberforce et al., 2016).

McCormack and McCance (2017) developed the Person‐centred Practice‐Framework (PCP‐Framework) to help healthcare staff gain a better understanding of the concept and to provide orientation to implement person‐centred care into practice. This framework is composed of five constructs: the macro‐context, the prerequisites, the care environment, the person‐centred processes and the person‐centred outcomes. These constructs cover the key aspects of the concept and are related to each other. First, the prerequisites, which represent the attributes of the staff members, must be considered in the context of developing an effective care environment. The person‐centred processes focus on care interventions to achieve person‐centred outcomes which are expected from effective person‐centred practice and which represent the core of the theoretical framework.

The successive promotion and the effective implementation of person‐centred practice is a process which is in constant movement and has to be evaluated regularly (De Silva, 2014). The evaluation should consider various perspectives (service users, staff, relatives) and diverse approaches (e.g., questionnaire, interview, and observation) to guarantee an overall evaluation of person‐centred practice (De Silva, 2014; Slater, McCance, & McCormack, 2017). However, most of the existing instruments measuring person‐centred practice focus on the patient's perspective or a proxy‐perspective provided by a care partner. Little has been found in the literature about instruments considering the perspectives of staff and how they experience person‐centred practice, although they are continuously and daily in contact with it (De Silva, 2014; Martinez et al., 2016). Besides, Slater et al. (2017) argue that the contextual and physical factors that concern the staff members represent the biggest challenge in implementing and promoting person‐centred practice. Furthermore, most instruments assess person‐centred outcomes or focus on one single aspect of the concept rather than on the concept per se (Han, 2016). Edvardsson and Innes (2010) criticized the current state of evaluation in person‐centredness, arguing that most existing instruments are not underpinned by a theoretical framework.

The *Person‐centred Practice Inventory for Staff *(*PCPI‐S*), developed by Slater et al. (2017), gives an opportunity to measure important elements of culture and context that contribute to the development of person‐centred practice using the PCP‐Framework as a guide. The instrument facilitates the implementation of person‐centred practice as it allows the evaluation and identification of practice domains that are person‐centred and those that need further improvement and development (Slater et al., 2017).

### 2.1 Description of the PCPI‐S

The PCPI‐S was developed by Slater et al. (2017) and aims to measure person‐centred practice and how staff perceive the concept. It consists of 17 dimensions with 59 items formulated as statements about the three constructs of prerequisites, the care environment and the person‐centred processes. Participants are asked to evaluate the statements on a five‐point Likert scale ranging from “strongly disagree” to “strongly agree” (Slater, McCance, & McCormack, 2015).

The instrument development was conducted in two phases (Slater et al., 2017). Phase 1 consisted of two Delphi studies to compare and find common definitions and items to measure the constructs of the PCP‐framework. In phase 2, the resulting questionnaire was tested in a quantitative cross‐sectional survey. The psychometric properties were good, and the instrument affirmed the structure of the theoretical framework. The PCPI‐S was recently translated and has been evaluated for psychometric properties in Norway by Bing‐Jonsson et al. (2018), who found acceptable psychometric properties as well.

Person‐centred practice has started to attract attention in the German‐speaking countries (Grossmann, Schäfer, van Lieshout, & Frei, 2018). However, no instrument was identified to evaluate person‐centred practice from staff member's perspective that includes all of the three aforementioned constructs. As the framework is being used more commonly in the German‐speaking countries to inform the development of person‐centred practice, a translation of the PCPI‐S to evaluate the effectiveness of person‐centred practice seems necessary. This will also provide the opportunity to compare results on an international basis.

## 3 OBJECTIVES

This article reports on the translation, the cultural adaptation and the psychometric evaluation of the German version of the PCPI‐S. Statistical analysis focusses on the evaluation of the construct validity and internal consistency. Furthermore, the article analyses whether the instrument affirms the theoretical PCP‐framework.

## 4 DESIGN AND METHODS

A two‐stage research design was used involving the translation and cultural adaption of the PCPI‐S from English to German (phase 1) followed by a quantitative cross‐sectional survey based on psychometric analysis (phase 2).

### 4.1 Phase 1: The translation process

The PCPI‐S was translated into German using the internationally approved principles of good practice in translation and cultural adaptation by the International Society for Pharmacoeconomics and Outcome Research (ISPOR; Wild et al., 2005). This guideline was used as an orientation during the translation process. However, no back translation was undertaken as there is uncertainty about the additional value of this procedure in the literature (Epstein, Osborne, Elsworth, Beaton, & Guillemin, 2015; Swaine‐Verdier, Doward, Hagell, Thorsen, & McKenna, 2004). Instead, Epstein et al. (2015) recommend a focus on a high‐quality forward translation. The PCPI‐S was renamed PCPI‐S (G‐LTC), with “G” standing for *German* and “LTC” as abbreviation for *long‐term care*.

### 4.2 Phase 2: Psychometric evaluation

Data collection was performed using a quantitative cross‐sectional survey to test the measurement model as found in the original version of the PCPI‐S (Slater et al., 2017). The German version was tested with a convenience sample of healthcare staff from 15 nursing homes. A total of 1,728 staff had the chance to participate in the survey. The sample was drawn from one organization in one state of a central European country, representing the long‐term care setting in the rural area. Clinical areas included older people care, hospice care, intensive care and psychosocial care. A standardized online survey was used simultaneously in these nursing homes to reach the recommended ratio of respondent to item of at least 5:1 (Bryant & Yarnold, 1995). The items were partitioned and analysed according to concepts (prerequisites, care environment and person‐centred processes) to maximize the item to respondent ratio.

Recruitment was undertaken through an existing cooperation between the Department of Nursing Science at the University of Vienna and the nursing homes. The staff were informed about the project by the directors of the nursing homes, who sent an email containing the URL to access the online questionnaire. Only staff members directly involved in the care process of the residents could participate, including registered nurses, therapists (physiotherapists, occupational therapists), nursing auxiliaries, social carers and assistants (without formal qualifications). Furthermore, the participants had to be fluent in the German language and be willing to participate. Anonymity and confidentiality of the data were assured.

Data were collected from 7–31 May 2018. The process of informed consent was made explicit by adding an information sheet to the email stating that a completed questionnaire indicates consent to participate. Staff members received a reminder via email to increase the participation rate on day 8 and day 15.

The study was conducted in line with ethical regulations in human research. The participants were informed about the research project, the nature of their participation, their right to withdraw at any time without repercussions in their work situation and their right to gain insight into the data and information about the findings. They were also informed about the strategies employed to keep their information confidential and to anonymize the data. All participants gave consent to participate in the study. For non‐interventional studies, approval from an ethical board is not necessary in Austria (because of the national regulations of research ethics in Austria).

### 4.3 Statistical analysis

For psychometric analysis, SPSS Statistics version 24 and SPSS AMOS version 25 were used to analyse the construct validity of the instrument. Confirmatory analysis was performed based on the assumed structure in accordance with the PCP‐Framework. Three confirmatory analyses (CFA) were computed as the questionnaire includes three main constructs (prerequisites, care environment, person‐centred processes) defined by different dimensions. Although strongly related to each other, the three constructs were analysed separately as they measure different aspects of person‐centredness from staff perspectives. If the construct validity of the three constructs is high, it can be assumed that the construct validity for person‐centredness is high, as the concept is defined by these three constructs. Internal consistency of the PCPI‐S (G‐LTC) was analysed by calculating Cronbach's *α*. Using CFA also enables the results to be linked to the theoretical measurement model (The PCP‐Framework).

As missing data can have an influence on the results of a CFA, only fully completed questionnaires were included in the analysis. Many items had skewness and kurtosis scores over the suggested limit value (skewness > 2, kurtosis > 7; West, Finch, & Curran, 1995). Therefore, the maximum‐likelihood robust (MLR) extraction was used in the CFA, as it is robust to categorical data and to items that are not normally distributed (Hu & Bentler, 1999). This method was also used in the original testing of the PCPI‐S (Slater et al., 2017). The Bollen‐Stine Bootstrap test needed to be applied as the data were not normally distributed to regularize the *p* value of *χ* ^2^ (Weiber & Mühlhaus, 2014). The presence of multivariate outliers was examined by the calculation of the Mahalanobis distance for each data case. Factor loadings greater than 0.3 were accepted (Weiber & Mühlhaus, 2014).

Modification indices were applied to the original theoretical model to improve the model fit, based on the same criteria that were fixed for the Norwegian translation: (a) correlated errors across items within the same factor; (b) correlated errors across items across factors; and (c) cross factor loadings of items to factors. Only modifications that are theoretically justifiable have been permitted. For instance, the correlation between item 14—“I use reflection to check out if my actions are consistent with my ways of being” and item 16—“I actively seek feedback from others about my practice” as it seems evident that a person who reflects on his/her own practices also appreciates and can handle feedback (positive or negative) more easily. If a person says that (s)he is open to feedback, (s)he must be or seem to be able to critique herself/himself or her/his practice.

The model was refined through a continuous and iterative process until it was considered acceptable, supported by a root mean square error of approximation (RMSEA) of 0.08 or below; a 90% higher bracket below 0.09; a Comparative Fit Index (CFI) of 0.90 or higher; and a standardized root mean square residual (SRMR) higher than 0.10 indicating a good model fit. Cronbach's alpha can be judged as acceptable under 0.7, as good between 0.7–0.9 and as excellent above 0.9 (Weiber & Mühlhaus, 2014).

## 5 RESULTS

### 5.1 Stage 1: Translation process

Permission for the translation of the PCPI‐S from English into German was obtained and the instrument developer agreed to be involved in the proceedings. First, two independent English linguistic experts, who were introduced to the concept of “person‐centredness,” translated the instrument into German and one translation was conducted by the first author. All the translators were fluent speakers in German and English. These three forward translations were first discussed independently with the first author, followed by a group discussion with the two forward translators, until consensus concerning the wording of the items was reached. This version was presented to three researchers with expertise in instrument development and gerontology, who evaluated the instrument concerning conceptual equivalence, wording, cultural relevance and comprehensiveness. Another group discussion followed to identify any discrepancies and to develop the final version of the PCPI‐S, which was eventually reviewed by a German language expert concerning wording, grammatical errors, spelling and punctuation errors.

In total, seven experts were involved in the translation process. In accordance with the setting, the term “patient” was changed to “resident” to adapt the instrument to the long‐term care setting, representing the more common term used in this specific setting. Two items of the original instrument were perceived as representing two different questions. These were item 40 “My organization recognizes and rewards success” (an organization which recognizes success doesn´t necessarily reward them) and item 43 “I have the opportunity to discuss my practice and professional development on a regular basis” (these are perceived as two different issues to discuss). Each of these items was separated into two items; hence, the PCPI‐S (G‐LTC) consists of 61 items compared with 59 items in the original version.

Finally, a pre‐test was performed by 15 experts and nursing professionals, including graduate students from both Master's and Doctoral programs at the Department of Nursing Science of the University of Vienna, 12 of whom were experienced nurses. The aim of this step was to evaluate the comprehensiveness and clarity of the instrument, as well as its ability to identify any lack of clarity concerning the answering of the questionnaire. Based on these results, some adaptations to the wording of various items were made. The conceptual equivalence, face validity and content validity were evaluated as “good” by the 15 experts.

### 5.2 Sample

A sample of 255 staff members of 15 nursing homes in Austria completed the 61 items of the PCPI‐S (G‐LTC). The sample consisted of 87% women and 13% men, of whom 36.1% were registered nurses, 41.6% nurse assistants and 22.3% with other qualifications (therapists, social carers, and assistants (without formal qualifications)). The mean length of work experience was 21.5 years (*SD* = 10.7; Range 2–46). Table 1 outlines the sample characteristics.

**Table 1:** Sample characteristics

Variable	*N*	%
Sex		
Female	220	86.3
Male	31	12.2
Age (years)		
<19	1	0.4
19–25	17	6.7
26–35	53	20.8
36–45	76	29.8
46–55	84	32.9
56–65	22	8.6
Profession		
Registered nurse	92	36.1
Therapist (physio, occupational)	8	3.1
Nurse assistant	106	41.6
Social carer	12	4.7
Other	31	12.2
Extent of employment		
≤75%	101	39.6
≥76%	152	59.6
Length of employment in year(s)		
<1	33	12.9
1–2	32	12.5
3–5	56	22.0
6–10	40	15.7
>10	90	35.3

Variable	Mean	Median	Standard Deviation (min‐max)
Experience (years)	21.5	22.5	10.7 (2–46)
Continued education past 12 months (hr)	33.7	18.0	78.8 (0–1,000)

Considering that the three constructs were analysed separately, the sample size represents a 14:1 ratio for the construct *prerequisites* with 18 items, a 9:1 ratio of respondents to items for the *care environment* with 28 items and a 15:1 ratio for the *person‐centred processes* with 17 items. This far exceeds the minimum required ratio of 5:1 and permits the obtaining of precise results in the CFAs (Bryant & Yarnold, 1995).

### 5.3 Stage 2: Psychometric testing

All items were positively scored showing mean scores ranging from 2.56–3.82 where 0 means “strongly disagree” and 4 means “strongly agree.” The examination of the data indicated skewness and kurtosis on many items, which is why the Bollen‐Stine Bootstrap method was applied for the three constructs. Factor loadings ranged from 0.172–0.890, whereas most of the factor loadings were over 0.5. Only four items failed to achieve acceptable factor loading of 0.3: v16, v21, v22 and v35. However, all the factor loadings were statistically significant (Standard error < 0.9; critical ratio > 1.96; *p* < .01) and showed a meaningful contribution to the measurement model, which is why these items were not excluded (Weiber & Mühlhaus, 2014). The factor loadings are set out in Table 2. The correlations between the factors were all positive and ranged from 0.31–0.97.

**Table 2:** Mean scores, measures of distribution and factor loadings of each item of the PCPI‐S (G‐LTC)

	Construct scores and items	Mean	Standard deviation	Factor loading	Standard error
Prerequisites (Cronbach's alpha)					0.902
Professionally competent (Cronbach's alpha)					0.687
v1	I have the necessary skills to negotiate care options	3.34	0.830	0.540	0.052
v2	When I provide care I pay attention to more than the immediate physical task	3.49	0.955	0.529	0.061
v3	I actively seek opportunities to extend my professional competence	3.40	0.797	0.611	0.048
Developed interpersonal skills (Cronbach's alpha)					0.828
v4	I ensure I hear and acknowledge others´ perspectives	3.45	0.766	0.553	0.044
v5	In my communication, I demonstrate respect for others	3.82	0.544	0.405	0.031
v6	I use different communication techniques to find mutually agreed solutions	3.60	0.673	0.561	0.036
v7	I pay attention to how my non‐verbal cues impact on my engagement with others	3.54	0.730	0.518	0.042
Being committed to the job (Cronbach's alpha)					0.857
v8	I strive to deliver high‐quality care to people	3.82	0.490	0.384	0.027
v9	I seek opportunities to get to know the person and their family in order to provide holistic care	3.60	0.625	0.426	0.036
v10	I go out of my way to spend time with people receiving care	3.52	0.714	0.494	0.041
v11	I strive to deliver high‐quality care that is informed by evidence	3.75	0.538	0.431	0.029
v12	I continuously look for opportunities to improve the care experiences	3.56	0.701	0.549	0.038
Knowing self (Cronbach's alpha)					0.814
v13	I take time to explore why I react as I do in certain situations	3.44	0.781	0.548	0.046
v14	I use reflection to check out if my actions are consistent with my ways of being	3.51	0.698	0.585	0.038
v15	I pay attention to how my life experiences influence my practice	3.50	0.663	0.528	0.037
Clarity of beliefs and values (Cronbach's alpha)					0.735
v16	I actively seek feedback from others about my practice	3.71	0.611	0.264	0.039
v17	I challenge colleagues when their practice is inconsistent with our team's shared values and beliefs	3.00	0.968	0.801	0.057
v18	I support colleagues to develop their practice to reflect the team's shared values and beliefs	3.35	0.842	0.730	0.050
The Care Environment (Cronbach's alpha)					0.941
Skill Mix (Cronbach's alpha)					0.633
v19	I recognize when there is a deficit in knowledge and skills in the team and its impact on care delivery	3.19	0.793	0.572	0.051
v20	I am able to make the case when skill mix falls below acceptable levels	3.16	0.860	0.677	0.056
v21	I value the input from all team members and their contributions to care	3.78	0.509	0.172	0.035
Shared decision‐making systems (Cronbach's alpha)					0.718
v22	I actively participate in team meetings to inform my decision‐making	3.66	0.613	0.266	0.039
v23	I participate in organization‐wide decision‐making forums that impact on practice	3.16	1.007	0.593	0.061
v24	I am able to access opportunities to actively participate in influencing decisions in my directorate/division	3.05	1.041	0.727	0.061
v25	My opinion is sought in clinical decision‐making forums (e.g., ward rounds, case conferences and discharge planning)	3.14	0.865	0.638	0.050
Effective staff relationships (Cronbach's alpha)					0.893
v26	I work in a team that values my contribution to person‐centred care	3.29	0.843	0.751	0.043
v27	I work in a team that encourages everyone's contribution to person‐centred care	3.32	0.881	0.750	0.046
v28	My colleagues positively role model the development of effective relationships	3.16	0.867	0.716	0.046
Power sharing (Cronbach's alpha)					0.860
v29	The contribution of colleagues is recognized and acknowledged	3.14	0.833	0.585	0.046
v30	I actively contribute to the development of shared goals	3.42	0.732	0.520	0.041
v31	The leader facilitates participation	3.02	1.008	0.805	0.053
v32	I am encouraged and supported to lead developments in practice	3.20	0.876	0.765	0.044
Potential for innovation and risk taking (Cronbach's alpha)					0.690
v33	I am supported to do things differently to improve my practice	3.13	0.946	0.838	0.050
v34	I am able to balance the use of evidence with taking risks	3.34	0.713	0.430	0.042
v35	I am committed to enhancing care by challenging practice	3.32	0.761	0.324	0.047
The physical environment (Cronbach's alpha)					0.797
v36	I pay attention to the impact of the physical environment on people's dignity	3.58	0.710	0.447	0.043
v37	I challenge others to consider how different elements of the physical environment impact on person‐centredness (e.g., noise, light and heat)	3.29	0.871	0.739	0.048
v38	I seek out creative ways of improving the pep environment	3.30	0.882	0.697	0.050
Supportive organizational systems (Cronbach's alpha)					0.903
v39	In my team, we take time to celebrate our achievements	2.56	1.162	0.700	0.068
v40	My organization recognizes and rewards success	2.97	1.031	0.787	0.056
v40a	/	2.28	1.241	0.890	0.069
v41	I am recognized for the contribution that I make to people having a good experience of care	3.04	0.951	0.789	0.049
v42	I am supported to express concerns about an aspect of care	2.91	1.014	0.868	0.052
v43	I have the opportunity to discuss my practice and professional development on a regular basis	2.98	0.980	0.773	0.052
v43a	/	2.83	1.028	0.782	0.056
Person‐centred Processes (Cronbach's alpha)					0.914
Working with patient beliefs and values (Cronbach's alpha)					0.728
v44	I integrate my knowledge of the person into care delivery	3.59	0.587	0.403	0.034
v45	I work with the person within the context of their family and carers	2.94	0.937	0.525	0.057
v46	I seek feedback on how people make sense of their care experience	3.60	0.580	0.447	0.032
v47	I encourage people to discuss what is important to them	3.71	0.504	0.371	0.029
Shared decision‐making (Cronbach's alpha)					0.695
v48	I include the family in care decisions where appropriate and/or in line with the person's wishes	3.07	0.967	0.637	0.058
v49	I work with the person to set health goals for their future	3.38	0.743	0.493	0.044
v50	I enable people receiving care to seek information about their care from other healthcare professionals	3.48	0.692	0.470	0.041
Engagement (Cronbach's alpha)					0.767
v51	I try to understand the person's perspective	3.66	0.593	0.476	0.032
v52	I seek to resolve issues when my goals for the person differ from their perspectives	3.58	0.652	0.552	0.035
v53	I engage people in care processes where appropriate	3.30	0.868	0.548	0.052
Having sympathetic presence (Cronbach's alpha)					0.787
v54	I actively listen to people receiving care to identify unmet needs	3.74	0.506	0.439	0.026
v55	I gather additional information to help me support people receiving care	3.57	0.641	0.419	0.037
v56	I ensure my full attention is focused on the person when I am with them	3.75	0.504	0.375	0.028
Providing holistic care (Cronbach's alpha)					0.864
v57	I strive to gain a sense of the whole person	3.78	0.466	0.384	0.024
v58	I assess the needs of the person, taking account of all aspects of their lives	3.64	0.586	0.480	0.031
v59	I deliver care that takes account of the whole person	3.74	0.507	0.428	0.026
Cronbach's alpha of the PCPI‐S (G‐LTC)					0.961

### 5.4 Modifications to the model

#### 5.4.1 Prerequisites

Correlated error between items v6 and v7; v8 and v11; v1 and v14; v14 and v16.

#### 5.4.2 The care environment

Correlated error between items v22 and v23; v29 and v31; v39 and v40; v39 and v40a; v40 and v40a; v40a and v41, v42 and v43; v43 and v43a; v32 and v33. Cross factor loading v21 on *Effective staff relationships*.

#### 5.4.3 Person‐centred processes

**Figure 1:**
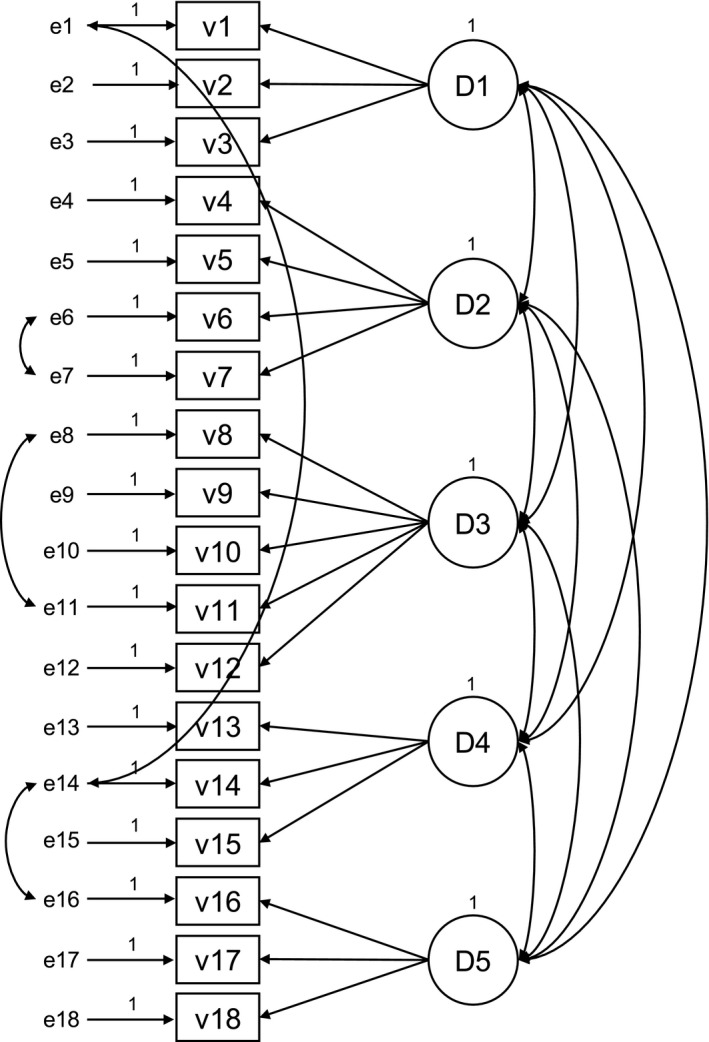
Refined measurement model “prerequisites”

**Figure 2:**
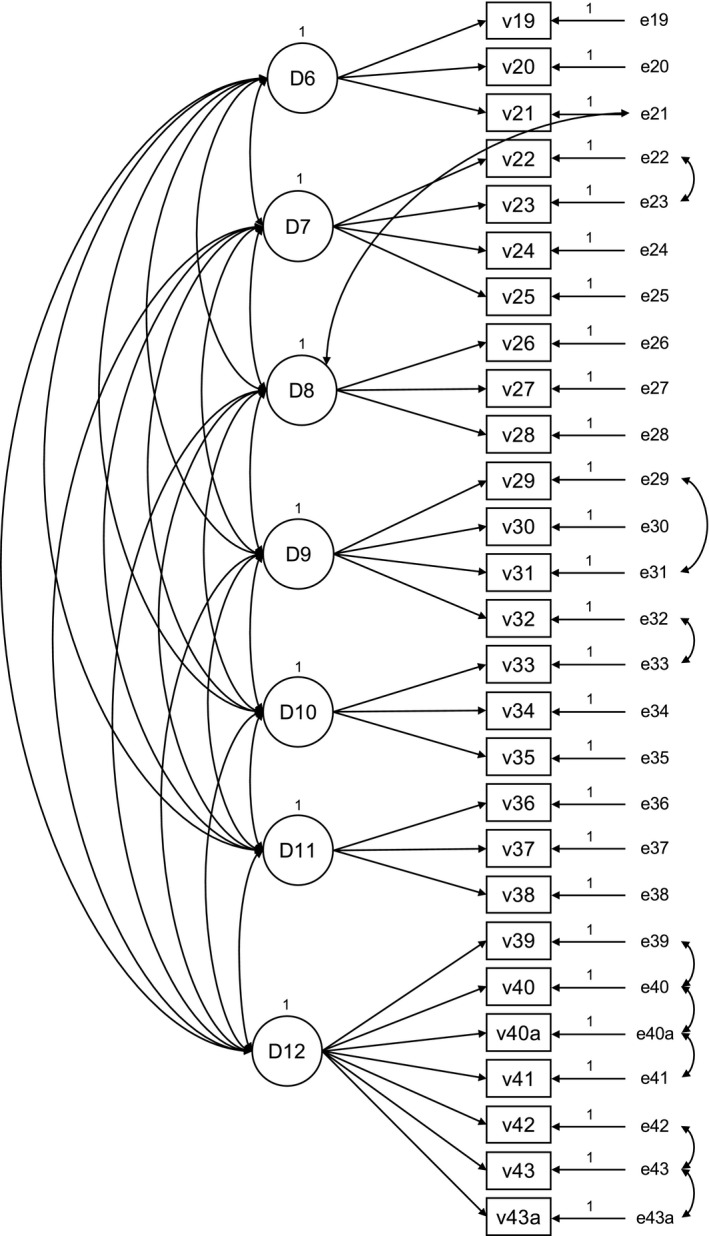
Refined measurement model “care environment”

**Figure 3:**
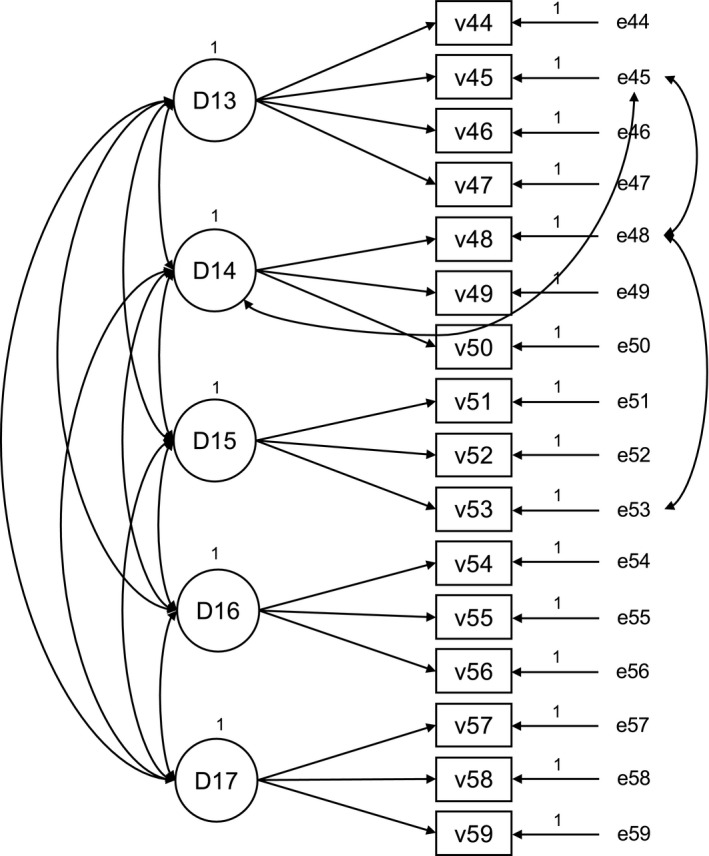
Refined measurement model “person‐centred processes”

Correlated error between items v45 and v48; v48 and 53. Cross factor loading v45 on *Shared decision‐making.* The Fit Statistics for the refined measurement models (Figures 1, 2, 3) of the PCPI‐S (G‐LTC) are set out in Table 3.

**Table 3:** Fit Statistics for refined measurement models of the PCPI‐S (G‐LTC)

Models	*χ* ^2^	*df*	χ2/*df*	CFI	RMSEA	90% RMSEA	SRMR
Prerequisites	232.3	121	1.920	0.948	0.060	0.048–0.072	0.0591
The care environment	680.1	293	2.321	0.907	0.072	0.065–0.079	0.0609
Person‐centred processes	187.6	91	2.062	0.957	0.065	0.051–0.078	0.0467

Item 21 (“I value the input from all team members and their contributions to care”) attracted attention during CFA. The factor loading to the dimension *Appropriate Skill Mix* failed to achieve the recommended score and a high correlation was identified with the dimension *Effective staff relationships*. The item relationship was significant for both factors, which is why it was maintained on its original factor.

#### 5.4.4 Cronbach's alpha

Cronbach's alpha scores for each of the constructs were acceptable and ranged from 0.633 for the factor *Appropriate Skill Mix* to 0.903 for *Supportive organizational systems*. The Cronbach's alpha scores for each factor are included in Table 2. Cronbach's alpha scores were excellent with a score of 0.902 for the construct prerequisites, 0.941 for the care environment and 0.914 for the person‐centred processes. The total scale of the PCPI‐S (G‐LTC) reached a score of 0.961, which is also excellent.

### 5.5 Summary of the psychometric evaluation

The model Fit Statistics of the three constructs indicate good model fit with a RMSEA of 0.08 or below; a 90% higher bracket below 0.09; a CFI of 0.90 or higher; and a SRMR higher than 0.10 indicating a good model fit. The detailed scores are set out in Table 3.

## 6 DISCUSSION AND IMPLICATIONS

The results of the psychometric analysis of the PCPI‐S (G‐LTC) present good construct validity and reliability and affirm the theoretical PCP‐Framework. The regular evaluation of the staff perceptions of person‐centred practice permits to assess the sustainability of the concept and to identify domains that need to be changed or improved (White, Newton‐Curtis, & Lyons, 2008). Furthermore, the evaluation enables staff members to critically reflect on their own practice, which in turn, recognizes the importance and significance of the concept (De Silva, 2014).

The three constructs (prerequisites, the care environment and person‐centred processes) can be measured and evaluated with the PCPI‐S (G‐LTC). However, the translation and cultural adaptation of instruments is a process that should not be underestimated. Next to the risk of construct distortion (cultural attitudes, taboo topics), the semantic and/or syntactical translation of the various items can be challenging in this process, which is why guidelines should be followed to ensure high quality and credibility of the results and a conceptual equivalence with the original instrument (Reuschenbach & Mahler, 2011).

The principles of good practice guideline by ISPOR (Wild et al., 2005), which consist of internationally approved recommendations, were used for this purpose. The developers of this guideline accentuate that the guideline should be considered an orientation rather than seen as stringent instructions. In the Norwegian translation (Bing‐Jonsson et al., 2018), the first back translation did not always convey the original meaning and some concepts of the instrument needed to be reviewed and clarified by the instrument developer. In our study, no back translation was performed as these have a high financial cost and time effort and the additional value of such a procedure remains controversial. An improvement of the conceptual equivalence or a higher quality of the translation has not been scientifically proven (Swaine‐Verdier et al., 2004). Epstein et al. (2015) compared the two methods (forward translation followed by back translation and forward translation without back translation) and concluded that a forward translation followed by a review by an expert committee showed better quality and a more accurate concept equivalence. In this study, the focus was placed on a high‐quality forward translation by including various experts in the process. Furthermore, the instrument developer was included in the process and was able to clarify some uncertainties in the items, which contributed to the conceptual equivalence to the original instrument.

Cross‐cultural surveys with an identical instrument in various languages permit the development of a strong evidence‐base relating to a concept, in the presented case the staff perceptions of person‐centred practice and the comparison of the data on an international level (Reuschenbach & Mahler, 2011).

Given the critique of Edvardsson and Innes (2010) that there was a lack of instruments based on theoretical frameworks, the PCPI‐S and its German and Norwegian equivalents represent advances in this area of work. The PCPI‐S measures multiple aspects of person‐centred practice and is not limited to one aspect as criticized by Han (2016). Moreover, the PCPI‐S covers the main aspects of person‐centredness and can therefore be used to measure the effectiveness of implementing person‐centredness, independent of the method used for this purpose (Slater et al., 2017). Another strength is that the PCPI‐S is appropriate for use in a variety of settings and among various healthcare professionals, which was affirmed by the authors who developed and tested the Norwegian version (Bing‐Jonsson et al., 2018).

Overall, the results of the psychometric evaluation of the PCPI‐S (G‐LTC) were good. Content validity and face validity have been approved by the 15 experts participating in the pre‐test. The model Fit Statistics were positive for the three constructs. Minor modifications were identified with the help of the modification indices and have been employed if theoretically justifiable. These were mostly confined to correlated errors, which were also present in the Norwegian translation (Bing‐Jonsson et al., 2018). Two cross‐factor loadings have been permitted to the model but did not change the factor structure of the models. These specifications in the model do not necessarily mean that the empirical data do not match the theoretical model, but rather confirm the affirmation by the authors of the PCP‐Framework that its different dimensions are strongly associated with each other.

Most of the items of the PCPI‐S (G‐LTC) were scored positively (see mean scores in Table 2), meaning that the staff members perceive their provided care as person‐centred. Further descriptive analysis is to be conducted to identify practice areas that need further development and/or improvement to compare the results with the findings of the original testing. Furthermore, even minor modifications made in the model imply a new psychometric analysis to make sure that these adaptations do not only represent characteristics of the sample.

As described before, item 21 (“I value the input from all team members and their contributions to care”) loaded on two factors but was kept in its original factor. This decision was taken only based on statistical values rather than considering the theoretical aspect. For further use of the PCPI‐S (G‐LTC), we recommend to place item 21 in the dimension *Effective staff relationships* subsequent to item 26 (“I work in a team that values my contribution to person‐centred care”), as the two other items in the original factor (v19 and v20) might influence the answering of item 21 of the participants.

To generalize the results, the instrument needs further psychometric validation on a larger sample size in long‐term care, as the sample was limited in this study. The PCPI‐S only covers three of the five constructs represented in the PCP‐Framework, which is why it should be combined with other measurements that consider the macro‐context and other perspectives (patient, relatives) of the person‐centred processes.

## 7 CONCLUSION

The psychometric analysis of the German PCPI‐S showed good results, indicating high construct validity and high reliability. The PCPI‐S (G‐LTC) is suitable for measuring attributes (prerequisites) of staff members working in complex organizational systems (the care environment) to provide effective care through person‐centred interventions (the person‐centred processes). Further testing on a larger sample is recommended to affirm its validity and reliability.

## CONFLICT OF INTEREST

We have no conflict of interest to declare.
